# Using data linkage to enhance the reporting of cancer outcomes of Aboriginal and Torres Strait Islander people in NSW, Australia

**DOI:** 10.1186/s12874-019-0884-8

**Published:** 2019-12-30

**Authors:** Hanna E. Tervonen, Stuart Purdie, Nicola Creighton

**Affiliations:** Cancer Institute NSW, PO Box 41, Alexandria, Sydney, NSW 1435 Australia

**Keywords:** Neoplasms, Indigenous, Australia, Data linkage

## Abstract

**Background:**

Aboriginal people are known to be under-recorded in routinely collected datasets in Australia. This study examined methods for enhancing the reporting of cancer incidence among Aboriginal people using linked data methodologies.

**Methods:**

Invasive cancers diagnosed in New South Wales (NSW), Australia, in 2010–2014 were identified from the NSW Cancer Registry (NSWCR). The NSWCR data were linked to the NSW Admitted Patient Data Collection, the NSW Emergency Department Data Collection and the Australian Coordinating Register Cause of Death Unit Record File. The following methods for enhancing the identification of Aboriginal people were used: ‘ever-reported’, ‘reported on most recent record’, ‘weight of evidence’ and ‘multi-stage median’. The impact of these methods on the number of cancer cases and age-standardised cancer incidence rates (ASR) among Aboriginal people was explored.

**Results:**

Of the 204,948 cases of invasive cancer, 2703 (1.3%) were recorded as Aboriginal on the NSWCR. This increased with enhancement methods to 4184 (2.0%, ‘ever’), 3257 (1.6%, ‘most recent’), 3580 (1.7%, ‘weight of evidence’) and 3583 (1.7%, ‘multi-stage median’). Enhancement was generally greater in relative terms for males, people aged 25–34 years, people with cancers of localised or unknown degree of spread, people living in urban areas and areas with less socio-economic disadvantage. All enhancement methods increased ASRs for Aboriginal people. The weight of evidence method increased the overall ASR by 42% for males (894.1 per 100,000, 95% CI 844.5–945.4) and 27% for females (642.7 per 100,000, 95% CI 607.9–678.7). Greatest relative increases were observed for melanoma and prostate cancer incidence (126 and 63%, respectively). ASRs for prostate and breast cancer increased from below to above the ASRs of non-Aboriginal people with enhancement of Aboriginal status.

**Conclusions:**

All data linkage methods increased the number of cancer cases and ASRs for Aboriginal people. Enhancement varied by demographic and cancer characteristics. We considered the weight of evidence method to be most suitable for population-level reporting of cancer incidence among Aboriginal people. The impact of enhancement on disparities in cancer outcomes between Aboriginal and non-Aboriginal people should be further examined.

## Background

Aboriginal people are known to be under-recorded in routinely collected datasets [1–3]. Reasons for under-recording are complex and include a lack of awareness and training to ask about Aboriginal status among health staff, and among Aboriginal people concerns about how the question was asked, racism and discrimination, privacy, a lack of cultural safety and difficulties in tracing identity [4]. Under-recording of Aboriginal status generally results in under-estimation of absolute measures of health indicators [5, 6].

It is possible to enhance reporting of health outcomes of Aboriginal people by linking data from several sources [7]. For example, Randall and colleagues showed that different enhancement methods using linked data increased the number of hospital admissions for Aboriginal people with varying impacts on admission and mortality ratios [6]. Several different methods for enhancing identification of Aboriginal people have been used, with no consensus on the optimal method. Australian guidelines on data linkage related to Aboriginal people recommend comparing the impact of several methods and choosing the optimal method based on the purpose of the analysis and characteristics of the datasets [7].

Aboriginal people are under-recorded in the New South Wales Cancer Registry (NSWCR) despite increased recording of Aboriginal status over time [3]. In the early 1980s, more than 80% of people on the NSWCR had unknown Aboriginal status, which had dropped to approximately 13% by 1999. A previous study examining the feasibility of enhancement of reporting of Aboriginal people using linked data from several data sources, including NSWCR, found that the number of cancer cases, and hence cancer incidence, for Aboriginal people increased following enhancement [2].

Estimates of health outcomes among Aboriginal people and the size of disparities compared with non-Aboriginal people can change depending on how Aboriginal status is reported and which enhancement method is used [5, 6]. Accurate and complete recording of Indigenous status is needed to reliably measure cancer outcomes, identify disparities and produce information about cancer among Indigenous people globally. Cancer registries are a key source of information for reporting cancer outcomes yet there are very few studies examining the impact of under-recording of Indigenous status on cancer incidence [8]. This study examined the impact of linked data enhancement methods on the number of cancer cases and cancer incidence rates among Aboriginal people in NSW, Australia, using common algorithms and population-based datasets.

## Methods

### Study design and data sources

This was a retrospective cohort study using linked routinely-collected health data. All cases of invasive cancer diagnosed and recorded in the NSWCR between 2010 and 2014 were included in the analyses. The NSWCR is a statutory population-based cancer registry which collects information about all invasive cancers diagnosed in NSW, Australia. Information about Aboriginal and Torres Strait Islander status in the NSWCR comes from multiple sources, such as hospital treatment episodes and death registration [3]. Pathology reports do not include information about Aboriginal and Torres Strait Islander status and, therefore, this information is missing if the NSWCR only receives a pathology notification. The NSWCR uses a progressive positive identification algorithm with a single notice from any source indicating a person to be Aboriginal or Torres Strait Islander taking precedence over any other information. Aboriginal and Torres Strait Islander status is assigned at a person level, rather than individual cancer case level. Torres Strait Islander people are included with Aboriginal people throughout this study due to the small number of people from the Torres Strait Islands residing in NSW and in recognition that Aboriginal people are the original inhabitants of NSW [4].

The NSWCR data were linked to the NSW Admitted Patient Data Collection (APDC), the NSW Emergency Department Data Collection (EDDC) and the Australian Coordinating Registry Cause of Death Unit Record File (COD URF). The APDC includes records of all hospital admissions in NSW public and private hospitals and day procedure centres, the EDDC includes information on presentations to emergency departments of public hospitals in NSW, and the COD URF includes information about deaths occurring in NSW. Data linkage was performed by the Centre for Health Record Linkage (CHeReL). The CHeReL uses Choicemaker software to perform probabilistic linkage of personal identifiers using a privacy-preserving protocol (http://www.cherel.org.au). The datasets used in this study are in the CHeReL’s Master Linkage Key. The CHeReL implements quality assurance procedures and performs clerical review of a sample of records to keep the estimated false positive and false negative linkage rate to less than 5 per 1000. The CHeReL provided a unique and arbitrary “Project Person Number” which enabled the records in each study dataset to be joined for an individual without the researchers accessing personal identifiers.

The APDC data covered a period between July 2001 and December 2017, the EDDC between January 2005 and December 2017, and the COD URF between January 1985 and December 2015. Aboriginal status is self-reported in the APDC and EDDC and is provided by the next-of-kin in the COD URF. Population data were based on data from the Australian Bureau of Statistics and obtained through the Secure Analytics for Population Health Research and Intelligence (SAPHaRI) data warehouse (Centre for Epidemiology and Evidence, NSW Ministry of Health).

This project was approved by the NSW Population and Health Services Research Ethics Committee (HREC/15/CIPHS/15) and the Aboriginal Health and Medical Research Council Ethics Committee (HREC Ref. No. 1201/16). Subject matter advice and Aboriginal community input was sought from the Cancer Institute NSW Aboriginal Advisory Group.

### Enhancement methods

The following methods for enhancing the reporting of cancer among Aboriginal people were used: ‘ever reported as Aboriginal’ [7], ‘Aboriginal on most recent record’ [7], ‘weight of evidence’ [2] and ‘multi-stage median’ [9] (Table 1). These methods were selected because they are among the most commonly used methods, represent a combination of simple and complex enhancement methods and are likely to provide a range of estimates. If a person was recorded as Aboriginal on the NSWCR or on the COD URF, a person was considered to be Aboriginal in the analyses. Our aim was to correct for under-recording of Aboriginal people in the NSWCR, so we only considered changing the status of those recorded as non-Aboriginal or with unknown status in the NSWCR. We considered the risk of a person being wrongly identified as Aboriginal in the COD URF to be low since the information is provided by the next-of-kin. Otherwise the four enhancement methods were applied to the data according to the descriptions provided in Table 1.

**Table 1:** The enhancement methods used in the analyses

Method	Description
Ever reported [7]	Recorded as being Aboriginal at least once in any of the data sources.
Most recent record [7]	Recorded as being Aboriginal in the most recent record in any of the data sources.
Weight of evidence [2]	Recorded as Aboriginal if 1) there are three or more units of information and at least two indicate that the person is Aboriginal; 2) if there are one or 2 units of information and at least one identifies the person as Aboriginal.
Multi-stage median [9]	The weight of evidence method is applied in a two-step process: firstly to each dataset individually; and then treating the results for each dataset as units of information.

### Statistical analysis

The number, proportion and characteristics of cases reported as Aboriginal using the NSWCR information and the four enhancement methods were compared. Characteristics considered in this study were: sex, age at diagnosis, year of diagnosis, cancer site, degree of spread (localised, regional, distant, unknown) [10], residential remoteness (major cities, inner regional, outer regional, remote/very remote) [11], and area-based socio-economic disadvantage (Index of Relative Socio-economic Disadvantage quintiles) [12]. For descriptive analyses, cancer sites were classified using clinical cancer grouping [13].

Age-standardised cancer incidence rates (ASR) were calculated for non-Aboriginal and Aboriginal people using the NSWCR Aboriginal status variable before enhancement. Cases with unknown Aboriginal status were considered non-Aboriginal. For Aboriginal people, cancer incidence was also calculated using the variables created by the four enhancement methods. Direct age-standardisation was calculated using the 2001 Australian standard population and NSW population data based on data from the Australian Bureau of Statistics [14]. Results were reported as rates per 100,000 with 95% confidence intervals (CIs) for all cancers and for the following sites: (female) breast (International Statistical Classification of Diseases and Related Health Problems, 10th Revision, Australian Modification code C50), colorectal (C18-C20), prostate (C61), lung (C34), melanoma (C43), and cervical cancer (C53).

The impact of different enhancement methods on the number of cases and on ASRs was examined in relative terms (% increase compared with the NSWCR variable). Analyses were performed using SAS Version 9.4 (SAS Institute, Cary, NC).

## Results

Overall 204,948 cases of invasive cancer were diagnosed in NSW in 2010–2014. Of these, 2703 (1.3%) were diagnosed among Aboriginal people based on the NSWCR Aboriginal status variable. There were 28,572 cases of cancer with unknown Aboriginal status (13.9%). After enhancement, the number of cases among Aboriginal people increased to 4184 (2.0%, ‘ever’), 3257 (1.6%, ‘most recent’), 3580 (1.7%, ‘weight of evidence’) and 3583 (1.7%, ‘multi-stage median’). The majority of cancer cases with a status change after enhancement were originally recorded as non-Aboriginal, rather than unknown Aboriginal status. For example, of the 877 cases of cancer with a status enhanced to Aboriginal using the weight of evidence method, 74% (*n* = 651) were recorded as non-Aboriginal and 26% (*n* = 226) had unknown Aboriginal status on the NSWCR.

Relative enhancement (per cent increase) was generally greater for males, people aged 25–34 years, people with cancers of unknown or localised degree of spread, people living in urban areas and areas with less socio-economic disadvantage (Table 2).

**Table 2:** Impact of enhancement on the number of cancer cases and relative increase (%) among Aboriginal people by demographic and cancer characteristics, 2010–2014

	NSWCR ^a^		Ever reported			Most recent record			Weight of evidence			Multi-stage median		
	n	%	n	%	Increase (%) ^b^	n	%	Increase (%) ^b^	n	%	Increase (%) ^b^	n	%	Increase (%) ^b^
Sex														
Female	1329	1.5	1920	2.1	44.5%	1575	1.7	18.5%	1689	1.9	27.1%	1701	1.9	28.0%
Male	1374	1.2	2264	2.0	64.8%	1682	1.5	22.4%	1891	1.7	37.6%	1882	1.6	37.0%
Age at diagnosis														
0–14	49	4.4	61	5.5	24.5%	54	4.9	10.2%	57	5.1	16.3%	56	5.1	14.3%
15–24	47	3.0	68	4.4	44.7%	61	3.9	29.8%	62	4.0	31.9%	64	4.1	36.2%
25–34	86	2.0	143	3.4	66.3%	120	2.8	39.5%	128	3.0	48.8%	130	3.0	51.2%
35–44	224	2.2	322	3.2	43.8%	276	2.8	23.2%	289	2.9	29.0%	293	2.9	30.8%
45–54	521	2.2	727	3.0	39.5%	627	2.6	20.3%	661	2.8	26.9%	675	2.8	29.6%
55–64	714	1.6	1076	2.4	50.7%	867	1.9	21.4%	949	2.1	32.9%	945	2.1	32.4%
65–74	660	1.2	1058	1.9	60.3%	785	1.4	18.9%	878	1.6	33.0%	876	1.6	32.7%
75–84	332	0.8	570	1.3	71.7%	388	0.9	16.9%	451	1.0	35.8%	447	1.0	34.6%
85+	70	0.4	159	0.8	127.1%	79	0.4	12.9%	105	0.6	50.0%	97	0.5	38.6%
Year of diagnosis														
2010	497	1.3	766	1.9	54.1%	594	1.5	19.5%	664	1.7	33.6%	661	1.7	33.0%
2011	532	1.3	830	2.1	56.0%	639	1.6	20.1%	704	1.7	32.3%	705	1.7	32.5%
2012	535	1.3	836	2.0	56.3%	646	1.6	20.7%	710	1.7	32.7%	712	1.7	33.1%
2013	569	1.4	851	2.0	49.6%	683	1.6	20.0%	745	1.8	30.9%	747	1.8	31.3%
2014	570	1.4	901	2.1	58.1%	695	1.7	21.9%	757	1.8	32.8%	758	1.8	33.0%
Clinical cancer group														
Skin	91	0.4	284	1.3	212.1%	177	0.8	94.5%	212	1.0	133.0%	216	1.0	137.4%
Head and neck	145	2.6	205	3.6	41.4%	171	3.0	17.9%	186	3.3	28.3%	184	3.2	26.9%
Upper gastrointestinal	340	2.1	442	2.7	30.0%	367	2.3	7.9%	394	2.4	15.9%	392	2.4	15.3%
Colorectal	299	1.2	442	1.8	47.8%	356	1.4	19.1%	385	1.5	28.8%	379	1.5	26.8%
Respiratory	430	2.2	553	2.8	28.6%	464	2.4	7.9%	508	2.6	18.1%	502	2.6	16.7%
Bone and connective tissue	25	1.7	34	2.3	36.0%	29	2.0	16.0%	32	2.2	28.0%	33	2.2	32.0%
Breast	314	1.2	472	1.9	50.3%	391	1.5	24.5%	413	1.6	31.5%	420	1.7	33.8%
Gynaecological	179	2.1	249	2.9	39.1%	207	2.4	15.6%	222	2.6	24.0%	223	2.6	24.6%
Urogenital	416	0.9	803	1.8	93.0%	547	1.2	31.5%	627	1.4	50.7%	633	1.4	52.2%
Eye and neurological	45	1.4	68	2.2	51.1%	51	1.6	13.3%	56	1.8	24.4%	57	1.8	26.7%
Thyroid and other endocrine	64	1.2	109	2.1	70.3%	81	1.5	26.6%	93	1.8	45.3%	94	1.8	46.9%
Lymphohaematopoietic	258	1.2	401	1.9	55.4%	313	1.5	21.3%	344	1.6	33.3%	343	1.6	32.9%
Ill-defined and unknown primary sites	97	1.9	122	2.3	25.8%	103	2.0	6.2%	108	2.1	11.3%	107	2.1	10.3%
Degree of spread														
Localised	819	1.0	1467	1.8	79.1%	1086	1.3	32.6%	1209	1.5	47.6%	1212	1.5	48.0%
Regional	668	1.5	964	2.2	44.3%	767	1.8	14.8%	857	2.0	28.3%	850	2.0	27.2%
Distant	661	2.2	783	2.6	18.5%	686	2.3	3.8%	713	2.3	7.9%	713	2.3	7.9%
Unknown	555	1.1	970	2.0	74.8%	718	1.5	29.4%	801	1.6	44.3%	808	1.7	45.6%
Remoteness														
Major Cities	1206	0.9	1998	1.4	65.7%	1472	1.1	22.1%	1639	1.2	35.9%	1633	1.2	35.4%
Inner Regional	831	1.7	1277	2.6	53.7%	1001	2.0	20.5%	1098	2.2	32.1%	1109	2.2	33.5%
Outer Regional	530	3.4	738	4.7	39.2%	620	4.0	17.0%	678	4.3	27.9%	675	4.3	27.4%
Remote/ very remote	136	12.7	171	15.9	25.7%	164	15.3	20.6%	165	15.4	21.3%	166	15.5	22.1%
Socio-economic disadvantage quintile^c^														
Q1: Least disadvantaged	131	0.3	280	0.7	113.7%	157	0.4	19.8%	194	0.5	48.1%	195	0.5	48.9%
Q2	352	0.9	596	1.6	69.3%	441	1.1	25.3%	487	1.3	38.4%	485	1.3	37.8%
Q3	474	1.1	785	1.9	65.6%	580	1.4	22.4%	650	1.6	37.1%	654	1.6	38.0%
Q4	843	1.8	1219	2.6	44.6%	1004	2.2	19.1%	1082	2.3	28.4%	1087	2.3	28.9%
Q5: Most disadvantaged	903	2.4	1304	3.4	44.4%	1075	2.8	19.0%	1167	3.1	29.2%	1162	3.1	28.7%

^a^NSWCR: Aboriginal status variable in the NSW Cancer Registry

^b^Relative increase compared with the number of cases based on the NSW Cancer Registry Aboriginal status variable

^c^Index of Relative Socio-economic Disadvantage

**Fig. 1:**
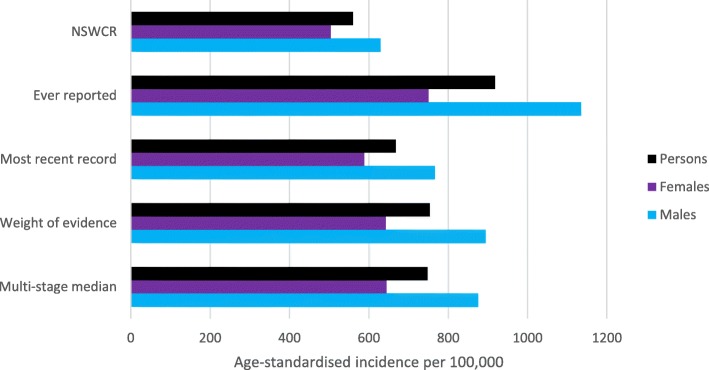
Age-standardised cancer incidence rates among Aboriginal people using the NSW Cancer Registry (NSWCR) Aboriginal status variable and four enhancement methods, 2010–2014. (see Table 3 for underlying data and 95% confidence intervals)

Overall the ASR among Aboriginal people was 559.9 per 100,000 (95% CI 535.3–585.3) before enhancement. All enhancement methods increased ASRs overall and for both males and females (Table 3, Fig. 1). The greatest increases were detected when using the ‘ever reported’ and the smallest increases when using the ‘most recent’ method. Enhancement increased incidence rates more for males than females. For example, the ‘weight of evidence’ method increased the ASR by 42% for males (894.1 per 100,000, 95% CI 844.5–945.4) and 27% for females (642.7 per 100,000, 95% CI 607.9–678.7).

**Table 3:** Age-standardised cancer incidence rates among non-Aboriginal and Aboriginal people, 2010–2014

	Non-Aboriginal people	Aboriginal people								
	NSWCR^a^	NSWCR^a^	Ever reported		Most recent record		Weight of evidence		Multi-stage median	
	ASR (95%CI)	ASR (95%CI)	ASR (95%CI)	Increase (%) ^b^	ASR (95%CI)	Increase (%) ^b^	ASR (95%CI)	Increase (%) ^b^	ASR (95%CI)	Increase (%) ^b^
All cancers										
Persons	490.8 (488.7–493.0)	559.9 (535.3–585.3)	918.0 (885.2–951.6)	64.0	667.9 (641.1–695.3)	19.3	753.7 (724.8–783.4)	34.6	748.0 (719.4–777.4)	33.6
Females	417.7 (415.0–420.5)	504.6 (474.0–536.5)	750.5 (712.2–790.1)	48.7	588.5 (555.7–622.6)	16.6	642.7 (607.9–678.7)	27.4	645.2 (610.4–681.2)	27.9
Males	577.6 (574.2–581.0)	629.4 (588.7–671.7)	1135.3 (1077.0-1195.5)	80.4	766.7 (722.0–813.0)	21.8	894.1 (844.5–945.4)	42.1	875.6 (827.3–925.7)	39.1
Colorectal										
Persons	57.8 (57.1–58.6)	63.9 (55.3–73.2)	104.5 (92.9–117.0)	63.5	77.7 (68.1–88.0)	21.6	86.3 (76.1–97.4)	35.1	84.1 (74.1–94.9)	31.6
Females	48.9 (47.9–49.8)	60.7 (49.8–73.0)	87.3 (73.7–102.3)	43.8	70.5 (58.7–83.7)	16.1	76.2 (63.9–90.0)	25.5	76.7 (64.2–90.6)	26.4
Males	68.1 (66.9–69.2)	67.3 (53.9–82.4)	127.7 (107.1–150.4)	89.7	86.3 (70.8–103.5)	28.2	99.7 (82.2–119.0)	48.1	92.7 (76.6–110.6)	37.7
Lung										
Persons	42.5 (41.8–43.1)	95.4 (85.2–106.3)	125.9 (113.9–138.6)	32.0	104.2 (93.4–115.7)	9.2	114.6 (103.3–126.7)	20.1	112.5 (101.4–124.4)	17.9
Females	33.3 (32.5–34.1)	83.9 (71.5–97.8)	103.8 (89.8–119.2)	23.7	89.8 (76.8–104.1)	7.0	97.5 (84.0–112.4)	16.2	96.8 (83.3–111.6)	15.4
Males	53.6 (52.6–54.7)	110.5 (93.6–129.2)	155.8 (134.7–178.7)	41.0	123.3 (105.1–143.4)	11.6	137.4 (118.1–158.7)	24.3	133.3 (114.5–154.0)	20.6
Melanoma										
Persons	50.8 (50.1–51.5)	16.7 (12.6–21.6)	57.3 (48.9–66.5)	243.1	28.4 (23.2–34.2)	70.1	37.8 (31.4–44.9)	126.3	36.8 (30.7–43.7)	120.4
Females	40.3 (39.4–41.2)	11.5 (7.1–17.2)	36.0 (27.7–45.6)	213.0	22.1 (16.3–29.0)	92.2	25.0 (18.4–32.9)	117.4	27.2 (20.3–35.4)	136.5
Males	63.4 (62.3–64.6)	23.3 (15.8–32.6)	85.0 (68.7–103.5)	264.8	36.1 (27.2–46.7)	54.9	53.5 (41.7–67.1)	129.6	47.7 (37.5–59.6)	104.7
Breast (Females)	120.8 (119.2–122.3)	105.2 (92.6–118.9)	161.1 (145.1–178.2)	53.1	130.9 (116.8–146.1)	24.4	140.0 (125.2–156.0)	33.1	142.1 (127.2–158.1)	35.1
Cervix	6.8 (6.4–7.2)	14.7 (10.5–19.9)	19.4 (14.5–25.3)	32.0	16.3 (12.0–21.6)	10.9	16.7 (12.3–22.1)	13.6	16.9 (12.5–22.3)	15.0
Prostate	167.7 (165.9–169.5)	126.1 (107.9–146.1)	281.8 (253.5–311.8)	123.5	172.7 (151.8–195.3)	37.0	205.2 (182.0–230.1)	62.7	203.6 (180.6–228.3)	61.5

ASR (95%CI): Age-standardised cancer incidence rate per 100,000 with 95% confidence intervals; directly standardised to the 2001 Australian standard population

^a^NSWCR: Aboriginal status variable available in the NSW Cancer Registry

^b^Relative increase compared with incidence rate based on the NSW Cancer Registry Aboriginal status variable

**Fig. 2:**
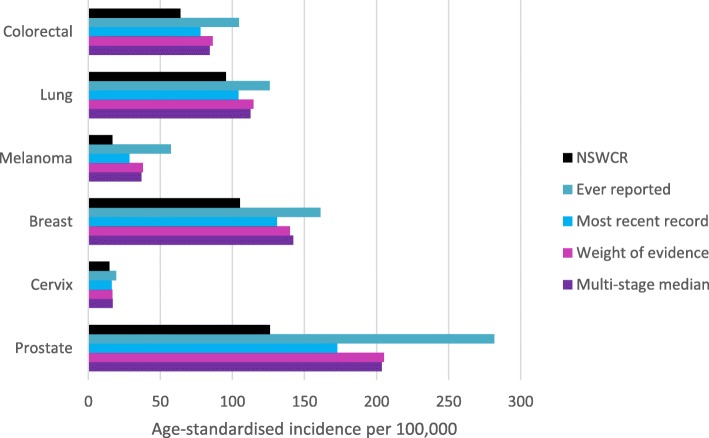
Age-standardised cancer incidence rates by site among Aboriginal people using the NSW Cancer Registry (NSWCR) Aboriginal status variable and four enhancement methods, 2010–2014. (see Table 3 for underlying data and 95% confidence intervals)

In site-specific analyses, all enhancement methods increased ASRs for all sites compared with rates estimated using the NSWCR Aboriginal status variable (Table 3, Fig. 2). Again, the ‘ever reported’ method demonstrated the greatest increases while the ‘most recent’ method resulted in the smallest increases. Greatest relative increases were observed for melanoma and prostate cancer incidence, with increases of 126 and 63% respectively, using the ‘weight of evidence’ method.

## Discussion

All enhancement methods increased both the number of cancer cases and age-standardised cancer incidence rates among Aboriginal people. The ‘ever reported’ method demonstrated the greatest increases and ‘most recent’ method the smallest increases, while the other two methods were very similar to each other and between these two extremities. When using the ‘weight of evidence’ method, the majority (74%) of cases with enhanced Aboriginal status were previously recorded as non-Aboriginal on the NSWCR. This indicates misclassification in the NSWCR Aboriginal status variable and highlights the need to correct this misclassification and not solely focus on decreasing the number of people with unknown Aboriginal status in the NSWCR and in the information received by the NSWCR from notifiers. Aboriginal and Torres Strait Islander status is self-reported at NSW health facilities and people may choose not to identify [4]. There have been strengthened procedures at a state level to improve the collection of Aboriginal and Torres Strait Islander status in NSW health facilities [15] as well as local initiatives to provide culturally safe health care throughout the study period. These factors are likely to have increased the willingness of people to self-identify as Aboriginal or Torres Strait Islander and improved identification at the point of care in more recent years. Linked data enhances the reporting of Aboriginal status because it brings together information on Aboriginal status that is not available to the NSWCR through people choosing to identify as Aboriginal after diagnosis or at facilities that have not provided cancer care.

Enhancement was generally greater in relative terms for males, people aged 25–34 years at diagnosis, people living in urban and less disadvantaged areas and for people with a cancer of localised or unknown degree of spread. Several factors are likely to explain these patterns, such as sources of cancer notifications and treatment patterns (e.g. the likelihood of admission for surgery). People diagnosed with cancers with good prognosis are less likely to be hospitalised or die which decreases the likelihood of recording the Aboriginal status on the NSWCR. If the NSWCR only receives pathology notification, Aboriginal status information will be missing. This is more likely to apply to cancers such as melanomas and prostate cancers, both of which showed greater levels of enhancement.

A previous NSW study reported that enhancing Aboriginal status for reporting deaths resulted in greater enhancements for older people, for females, for people living in urban areas and for those with chronic health conditions [16]. Another NSW study examining the impact on enhancement on hospital admissions reported greater enhancement for earlier years of admission, major cities, private hospitals and varying impact by age depending on the enhancement method used [6]. Different factors impact on enhancement depending on the health outcome of interest and the datasets used in analyses.

Lung and cervical cancers saw the smallest increases in incidence rates. Both these cancers have a greater burden in Aboriginal compared with non-Aboriginal people [17]. Due to the poor prognosis, death certificate information is available for most people diagnosed with lung cancer, increasing the likelihood of Aboriginal status recording. It is likely that enhancement had a smaller impact on lung cancer incidence rates because the existing NSWCR Aboriginal status already had relatively good capture. The relatively smaller increase in the incidence of cervical cancer may due to relatively good capture on the NSWCR, but may also be due to other factors such the patterns of hospitalisation and capture of Aboriginal status at the point of care for what is generally a younger cohort of women.

Enhancing the reporting of cancer outcomes of Aboriginal people might have a major impact on observed disparities between Aboriginal and non-Aboriginal people. For example, according to national statistics [17] and our analyses using the NSWCR Aboriginal status variable, Aboriginal people have lower breast and prostate cancer incidence rates compared with non-Aboriginal people. This pattern has also been reported among Indigenous peoples in many international jurisdictions and has been proposed as being related to the prevalence of risk factors for these cancers and competing causes of death [18]. After enhancement our results indicated higher breast and prostate cancer incidence among Aboriginal people than non-Aboriginal people in NSW. This finding has implications on widely held views on risk of these cancers among Indigenous peoples. Higher breast cancer rates have been reported among Indigenous people (Māori) in New Zealand using the national population-based cancer registry which includes links to a national health database to improve identification [18]. Increased breast cancer incidence among Indigenous people have been reported in two United States (US) states using data linkage between cancer registries and health service data [19, 20]. Our results also highlight the burden of melanoma among Aboriginal people which warrants further discussion on prevention strategies and actions. After enhancement our results indicated substantially higher incidence than when using the NSWCR Aboriginal status variable, but still lower rates compared with non-Aboriginal people (except when using the ‘ever reported’ method). The effect of under-recording of Indigenous status should be investigated in more jurisdictions. Cancer is the second leading cause of death and among the leading causes of burden of disease among Aboriginal people in Australia [21]. The findings of our study highlight the impact of cancer on Aboriginal people and the need for cancer control to improve health outcomes. Cancer control programs should have a special focus on Aboriginal people considering that their cancer burden may be higher than expected. Australian cancer screening programs are already targeting Aboriginal people due to lower participation rates [17].

Future research should also examine the impact of enhancement on other cancer outcomes, such as mortality, survival and the likelihood of being diagnosed with advanced stage disease. Studies have shown that Aboriginal people are more likely to be diagnosed with advanced stage cancer than non-Aboriginal people [22, 23]. We found greatest enhancement for people diagnosed with localised or unknown degree of spread, which may impact on the likelihood of Aboriginal people being diagnosed with advanced cancer in comparison with non-Aboriginal people and affect estimates of disparities in survival outcomes since localised cancers have much better prognosis.

Based on these results and consultation with the Cancer Institute NSW Aboriginal Advisory Group, the ‘weight of evidence’ method was considered to be the most suitable for further reporting of cancer outcomes for Aboriginal people. The ‘weight of evidence’ method utilises information from several sources but is still relatively straightforward to use and report. It provides a balance between enhancing the identification of Aboriginal people and reducing misclassification of non-Aboriginal people as Aboriginal. This method was developed and is also used by the NSW Ministry of Health [6]. Studies have pointed out that ‘ever reported’ may result in misclassification and over-reporting [1, 6]. It should be noted that an enhanced Aboriginal identifier is a statistical construct that enables improved reporting of cancer outcomes using historical data but potentially includes some inaccuracies due to errors in the source datasets and incorrect linkages [2]. Collection of accurate information at the point of care remains vital.

Limitations include that if a person was recorded as Aboriginal on the NSWCR or death certificate, this information was accepted. Although there is a possibility for positive misclassification this is likely to be low since the information is provided by the next-of-kin. Numerator-denominator bias is a known issue affecting observed cancer burden in Indigenous populations internationally because incidence and population data are derived using different data collection methodologies [8]. Population denominators can be unreliable due to under-participation of Aboriginal people and varying propensity to identify as Aboriginal in censuses. The Australian Bureau of Statistics (ABS) estimates Aboriginal and Torres Strait Islander populations using self-reported information in the Australian Census data with adjustment for undercount using a household survey following the census [14]. An increase in the number of people self-identifying as Aboriginal or Torres Strait Islander has been observed, with people who did not self-identify in the 2011 Australian Census choosing to identify in the subsequent 2016 Census [24]. In our study, enhancement of the numerator is likely to reduce the under-estimation of cancer incidence that is common in cancer incidence estimates for Indigenous people [8]. However, without enhancement of the denominator using the same methodologies it may lead to over-estimation of incidence rates. Linkage of the cancer registry, census, hospital and mortality data would enable cancer outcomes for Aboriginal people to be estimated with reduced numerator-denominator bias.

## Conclusions

All data linkage enhancement methods increased the number of cancer cases and cancer incidence rates for Aboriginal people. Enhancement varied by demographic and cancer characteristics. We considered the ‘weight of evidence’ method to be most suitable for future analyses of cancer outcomes of Aboriginal people. Enhancing the reporting of cancer outcomes of Aboriginal people can have major impacts on cancer disparities between Aboriginal and non-Aboriginal people and this should be further examined.

## Data Availability

Restrictions by the data custodians mean that the datasets are not publicly available or able to be provided by the authors. Researchers wanting to access the datasets used in this study should refer to the Centre for Health Record Linkage application process (www.cherel.org.au/apply-for-linked-data).

## Abbreviations

ABS: Australian Bureau of Statistics
APDC: Admitted Patient Data Collection
ASR: Age-standardised cancer incidence rate
CI: Confidence Intervals
COD URF: Cause of Death Unit Record File
EDDC: Emergency Department Data Collection
NSW: New South Wales
NSWCR: New South Wales Cancer Registry
US: United States
